# First District Dental Society of the State of New York

**Published:** 1886-01

**Authors:** 


### FIRST DISTRICT DENTAL SOCIETY OF THE STATE OF NEW YORK.

REPORTED EXPRESSLY FOR THE INDEPENDENT PRACTITIONER.

This society held its December meeting on the 4th ult., the President, Dr. W. Carr, in the chair.

Dr. W. H. Dwinelle referred to statements made at a previous meeting concerning the priority of the use of jack-screws for regulating teeth. He instanced an incident occurring about the year 1845, when a physician was called upon to perform an operation on a farmer who was injured while plowing in his field. During the excitement of the occasion the physician's case of instruments got mislaid. A search for them proved futile, but many months later they were found at some place in the field. Exposed to changes of weather the case became disjointed and the instruments quite rusty, with the exception of an amputating knife, the blade of which was secured to the handle with zinc. The appearance of the knife in so much better state of preservation than the other instruments led him to believe that galvanic action had prevented it from rusting. This suggested the idea that steel appliances could be used in the mouth. The jack-screw, being an instrument capable of great force, could be made of steel and adapted to the use of the dentist in moving the teeth. To prevent it from rusting, it was only necessary to drill a small hole in the appliance and load with zinc. He thinks jack-screws were not used by dentists prior to that period, and indeed could not have been used in the mouth, as they would have become useless from rust.

He also took the occasion to utter a word of warning against the indiscriminate use of cocaine. He said that many individuals used it freely without knowing much concerning its action, and he feared that mischief might result from its use.

*Dr. F. Y. Clark*—Had heard cocaine condemned and had read accounts of its injurious effects. He had also been anxious to learn if such reports were true, which he seemed to somewhat doubt. He believes it harmless, and has used it over five hundred times, and never with any ill result whatever. He claims that cocaine is now selling for nearly half its cost of manufacture, but soon the price will be high ; that parties will take advantage of stories concerning its danger and make a "corner" of it. He stated that the natives of countries where coca is found have used it freely for many years, and with them it is as common in use as tobacco.

Prof. E. T. Darby, of Philadelphia, the essayist for the evening, was introduced by the President, and produced a paper entitled "Erosion of Enamel and Etiology of Labial and Buccal Caries."

The paper stated that when caries first manifests itself near the gum, beginning with a green or brown stain, showing poor enamel, which readily becomes eroded, it is usually attributed to an abnormal condition of the buccal fluids which, having become acidulated, dissolve out the lime salts and render certain portions chalky. This he denominates "labial caries of childhood." The decay here is white, rapid in progress, appearing as if acids were acting upon the teeth. In such cases, treatment should be commenced as early as possible, and should be both local and constitutional. He would polish the eroded surfaces as thoroughly as possible, have the acid influences modified by the use of lime-water and prepared chalk, administer some preparation of hypophosphite of lime with cod liver oil, and advise plenty of health-giving exercise. For cavities thus formed he would use oxy-phosphate of zinc, which he considers the most suitable, or gutta-percha, which is next in value. Gold he would not recommend, as he believes it the worst material for such cavities. The plastic stoppings he would consider as temporary, and even gold, if used in such cases, would be sure to prove of mere temporary use. The main thing to be done is to arrest the trouble as far and completely as possible, and cases taken in time are easily arrested.

Dr. Darby referred to another condition of decay, found at the cervical border of the teeth, just beyond the termination of the enamel, or close to the margin of the gum. At this point the tooth substance softens or disintegrates, and is easily cut away. This occurs after puberty. It attacks buccal surfaces of molars, and sometimes bicuspids, but is rarely, if ever, found in front teeth. Recession of the gum is the first indication. It cannot be caused by the brush. There must be some other reason. Sometimes, where fillings have been introduced, the dentine disappears around the fillings, leaving them sticking out. He formerly considered this condition due to the action of acids, which dissolve away the surface, the brush removing the parts decalcified, but the highly polished appearance of the grooves found on the labial sufaces had somewhat changed his mind. Sometimes, most of the enamel surfaces have a gray and wasted appearance, and he wonders if it may not be due to a lack of lime-salts in the system, or a want of better nourishment, or, perhaps an excessive degree of calcification of tooth structure. He thinks the cases of buccal decay referred to are benefited by treating with the galvano-cautery, nitrate of silver or chloride of zinc. He would excavate and fill with gold. This, however, only protects the cavity already formed. If there is, apparently, a lack of lime salts, he would administer preparations of lime.* He considers a too free use of tobacco, especially cigarettes, as tending to aggravate this trouble. Long-standing cases of general debility and nervous prostration tend to induce it. Cavities thus formed must be filled, and if the mischief continues or extends beyond the fillings, larger fillings are needed; yet fillings do not always stay the trouble. Gutta-percha serves a good purpose, but gold is better, and with this material he would build up the injured parts of the teeth. The essayist referred to another kind of erosion found on the buccal and labial surfaces of teeth, and not resembling the cases before described. Very little has been published concerning it, and if referred to, no satisfactory explanations have appeared. Writers disagree in regard to it, and give different accounts concerning it. Tomes says it is not the result of too much brushing, for it is found at points where no brush could possibly reach, and in cases where no brush was ever used; also that grooves are found on the teeth of the sea lion in places where least used. This kind of erosion usually appears after the age of thirty. It begins on the incisors, by a wasting near the gum and the showing of grooves like cuts. Sometimes the entire surface seems to waste. These grooves are frequently highly polished, and yet extremely sensitive to thermal influences and sweets. Sometimes it is only on one side of the mouth, and there seems to stop. It is easier to see it than to ascertain its cause. If acids are used, or generated in the mouth, it does not prove that this condition is dependent on acid, for it is found where the saliva is alkaline or neutral. It does not seem to occur at a particular period. If caused by the brush, it is strange that it

*Rachitis is a lack of lime salts in the bones, in which the trophic changes are much greater than in the teeth. Can Dr. Darby cure this disease by the administration of any form of lime? Can he prescribe *any* course of treatment that may be safely recommended as inducing radical changes in the osseous system?—EDITOR.

is not found all around where the brush passes, and why so many who brush vigorously are exempt. He cited two cases that came under observation, which, in some respects, were similar, yet presenting different features of enamel erosion. Two ladies, of about forty years of age, had been his patients for over ten years. Each possessed good teeth, almost perfect. Both in good health, and neither used acid food. Each with good digestion, but the oral secretions abnormal. Both cases presented bad conditions of enamel erosion, never before suspected, yet commencing at about the same time. The conditions of the enamel and dentine were dissimilar, and the degree of sensitiveness differed.

*Dr. Dwinelle*—Remarked that the problem was still unsolved, and that the paper left it the same. He has endeavored to solve it many times, but had never succeeded. He referred to the case of a gentleman with teeth eroded across their cervical borders, and grooved as if done with a file, and highly polished. Within a year the grooves had extended to the pulp canals of several of the teeth. In endeavoring to account for this condition, he asked the gentleman if he had not taken into his system iodide of potash extensively. He received an affirmative reply. At that time Dr. Watt, of Ohio, then visiting New York, happened to be in his parlor, and was invited in to look at the case. His opinion being asked, he remarked that they had the appearance of being saturated with iodide of potash, thus confirming the view taken by Dr. D., as before expressed. Dr. Dwinelle thinks that our chemistry is constantly changing in this particular. Sometimes it is an acid, sometimes an alkali, and sometimes we say it is a deficiency of lime; but we can only speculate. A young lady called on him whose teeth had become much eroded in a few months' time, caused by a too free use of lemon juice. He smoothed and polished the eroded surfaces, and administered a preparation of lime. A year after he found that the trouble had been arrested.

*Dr. C. E. Francis*—Stated that the worst case of enamel erosion that ever came to his notice was in the mouth of a young lady, an invalid, who for a long time had been dosed with bromide of potash and bromide of soda. The erosion was so complete that scarcely a particle of enamel was left on her teeth, and they were extremely sensitive to the touch, and for use, worthless.

*Dr. C. F. W. Bödecker*—Had collected many such teeth, and prepared specimens for the microscope, in hopes to discover the secret, but the results were not altogether satisfactory. In his own mouth were two incisors somewhat denuded of enamel. He had tested the saliva for acid and alkali, but found neither. He uses chalk, also the galvano-cautery.

*Dr. F. Y. Clark*—It seems a mooted question whether or not erosion takes place on devitalized teeth. He had seen several cases that were thus affected. He was not ready to admit that it was a constitutional defect.

*Dr. Darby*—Remarked that he had also seen grooves on devitalized teeth, but presumed that the erosion took place before they became devitalized.

*Dr. J. W. Clowes*—Knew of no subject so vast and important. He used to hear of the denuding process, but no one seemed to know much about it. When he saw the old-time black decay, he knew what it meant and how to treat it, but now we have this terrible white decay, and we stand aghast! He contends that it is no riddle, but is in great part due to tonics prescribed by physicians. Excessive use of acids and alkalies act upon the teeth and cause them to melt away. He wishes that some impression could be made upon physicians that would cause them to desist. Such medicines baffle the efforts of the dentist to save teeth. In old times such was not the case, and it should not be so now. He uttered an earnest protest against giving tonics that destroy the teeth. The excuse for giving such remedies is that it is their duty to save life, even though the teeth are sacrificed. He does not believe in giving medicines that destroy one part to cure another. He had carefully cultivated good teeth only to have them riddled by erosion. But it is not a "mystery." The tincture of iron dissolves out the tooth structure, and the brush makes the grooves.

*Dr. W. T. Laroche*—Said that the best thing to do in cases alluded to was to polish and burnish the denuded parts.

*Dr Frank Abbott*—For many years thought he had the problem solved. He believed it caused by acids and the brush. Now he thinks they have not so much to do with it, but believes it in great part due to a congenital defect of enamel structure. He finds many cases of imperfect enamel with soft spots, or spots less calcified, where friction would have effect and cut ridges. He considers this a field for study.

